# Increased Circulating CD4^+^CXCR5^+^ Cells and IgG4 Levels in Patients with Myelodysplastic Syndrome with Autoimmune Diseases

**DOI:** 10.1155/2021/4302515

**Published:** 2021-09-30

**Authors:** Na Xiao, Xin He, Haiyue Niu, Hong Yu, Ningbo Cui, Hongzhao Li, Li Yan, Zonghong Shao, Limin Xing, Huaquan Wang

**Affiliations:** Department of Hematology, General Hospital, Tianjin Medical University, Tianjin, China

## Abstract

**Objectives:**

Immune abnormalities play an important role in the pathogenesis and progression of myelodysplastic syndrome (MDS). Some patients with MDS have autoimmune diseases (AI). Follicular helper T (Tfh) cells help B cells produce antibodies. The role of Tfh in MDS with AI has not been studied.

**Methods:**

We enrolled 21 patients with MDS with AI and 21 patients with MDS without AI. The proportion of peripheral blood CD4^+^CXCR5^+^ cells and the PD1 expression on CD4^+^CXCR5^+^ cells were detected by flow cytometry. Serum levels of immunoglobulin G (IgG) and IgG4 were measured. The survival and progression of MDS to acute myeloid leukemia (AML) in MDS patients with or without AI were compared.

**Results:**

MDS with AI accounted for 19.6% of all MDS cases in our study. The overall response rate was 81% (17/21) in MDS patients with AI for the first-line treatment. The proportion of circulating CD4^+^CXCR5^+^ cells was increased, but the expression of PD1 was decreased in MDS patients with AI. Serum IgG4 levels were also increased in MDS patients with AI. The proportion of peripheral blood CD4^+^CXCR5^+^ cells and the level of serum IgG4 decreased after therapy, but the expression of PD1 increased. There were no differences in overall survival and progress to acute myeloid leukemia between MDS with AI and without AI groups.

**Conclusion:**

CD4^+^CXCR5^+^ cells and IgG4 levels increased in patients with MDS and AI.

## 1. Introduction

Myelodysplastic syndromes (MDS) are a group of heterogeneous hematopoietic stem cell diseases. Anemia, neutropenia, and thrombocytopenia are the main clinical manifestations [1, 2]. Dysplastic development of hematopoietic stem and progenitor cells mainly affects myeloid cells but sometimes also partially affects lymphocytes. These dysplastic cells cause immune abnormalities, which may lead to autoimmune damage in some patients with MDS. Patients with MDS have obvious immune abnormalities, including cellular and humoral immunity, and defects in T cell and B cell functions. Dysplastic immune cell development leads to the most common and deadly complication of MDS. Dysplastic immune cells attack the normal hematopoietic system, leading to cytopenia, infection, bleeding, anemia, and even death. Abnormal immune cells cannot perform immune surveillance, and MDS may progress to acute myeloid leukemia (AML) [3, 4]. Approximately 11%-48% patients with MDS have autoimmune abnormalities [5–8].

The production of antibodies by B cells requires the help of T cells. Follicular helper T cells (Tfh) are T cell subsets with B cell helper functions and are one of the most common and important effector T cell subsets in lymphoid tissues. Tfh is significantly different from Th1 and Th2 cells. Its chemokine receptor, CXCR5, locates and migrates into B cell follicles. Tfh cells secrete the helper cytokine IL-21, which binds to IL-21R on B cells, leading to their differentiation into antibody-producing cells [9, 10]. An abnormal number of Tfh cells and subsequent expression of Tfh cell-related molecules may be related to the pathogenesis of some autoimmune or immunodeficiency diseases [11, 12].

This study investigated the number of circulating CD4^+^CXCR5^+^ cells and immunoglobulin levels in MDS patients with immune diseases.

## 2. Methods

### 2.1. Patient Characteristics

From September 2015 to June 2018, a total of 21 newly diagnosed MDS patients with autoimmune disease in the Hematology Department of the General Hospital of Tianjin Medical University were enrolled in the study, including 8 men and 13 women with a median age of 49 years (range 20-87 years) (details in Table 1).

Autoimmune diseases (AI) were diagnosed according to the international diagnostic criteria. Responses to the treatment of autoimmune diseases were evaluated based on the response criteria [13–17].

Twenty-one MDS patients without AI were selected as controls in this study, including 11 men and 10 women with a median age of 53 (range 21-83) years. Patients of similar sex, age, MDS subtyping, and gene mutation were selected as the control group, which made the results of the control group and the MDS-AI group comparable.

Informed written consent was obtained from all patients or their guardians according to the Helsinki Declaration. The study was approved by the Ethics Committee of the General Hospital of Tianjin Medical University.

### 2.2. Circulating CD4^+^CXCR5^+^ Lymphocyte Analysis with Flow Cytometry

Peripheral blood samples (3 mL) from MDS patients with AI and without AI were collected using heparin anticoagulant sterile tubes. The cells' forward scatter (FSC) and side scatter (SSC) were used to divide peripheral blood mononuclear cells into three subgroups: lymphocytes, monocytes, and granulocytes. The CD4-FITC monoclonal antibody and CXCR5-APC monoclonal antibody were used to define CD4^+^CXCR5^+^ cells and CD4^−^CXCR5^+^ cells. The expression of programmed cell death protein 1 (PD1) on CD4^+^CXCR5^+^ cells was measured using a PD1-PE monoclonal antibody. CD4-FITC, CXCR5-APC, PD1-PE, and isotype control monoclonal antibodies were purchased from BD Biosciences, USA. Data acquisition and analysis were performed using a FACSCalibur flow cytometer (BD Biosciences, USA) and Cell Quest software (Becton Dickinson, version 3.1) (supplemental data (available here)).

### 2.3. Serum Immunoglobulin G and Immunoglobulin G4 Tests

Serum levels of immunoglobulin G (IgG) and IgG4 were measured using an immune nephelometric analyzer with the BN™ II System (Siemens, Germany). Human immunoglobulin G4 N latex reagent, L2SUBM, L2PWSM, quality control, and standard products were purchased from Siemens, Germany. Detection was carried out according to the manufacturer's instructions.

### 2.4. Statistical Analysis

Results were analyzed using GraphPad Prism 8.0 (GraphPad Software, Inc. San Diego, CA). Data with normal distribution are presented as the means ± SD, and multiple group comparisons were performed using one-way analysis of variance (ANOVA). The index changes pre- and posttherapy were performed using a paired *t*-test. Overall survival analysis was performed using the log-rank test. The risk of progression to AML was determined using Fisher's exact test. Statistical significance was set at *P* < 0.05.

## 3. Results

### 3.1. Clinical Features of MDS Patients with AI

During this study period, we diagnosed a total of 107 MDS patients, of whom 21 (19.6%) were diagnosed with MDS with AI. MDS patients without AI (21 patients) were selected as controls.

The subtype of MDS patients with AI included three cases of MDS with single lineage dysplasia (MDS-SLD), six of MDS with multilineage dysplasia (MDS-MLD), seven of MDS with excess blasts 1 (MDS-EB1), and five of MDS with excess blasts 1 (MDS-EB2). According to the revised International Prognostic Score System (IPSS-R), two cases with very low score, five cases with low score, five cases with intermediate score, four cases with poor score, and 5 cases with very poor prognostic score were enrolled. There were no differences in age, sex, MDS subtype, and risk stratification between MDS patients with AI group and MDS patients without AI group (*P* > 0.05) (Table 1).

MDS patients with AI were classified as having systemic vasculitis, systemic lupus erythematosus, rheumatoid arthritis, psoriasis, Sjogren's syndrome, inflammatory bowel disease, Sweet's syndrome, Behcet's disease, Pyoderma gangrenosum, and granulomatosis with polyangiitis, in five, four, three, two, two, one, one, one, and one cases, respectively (Table 2).

### 3.2. Increased CD4^+^CXCR5^+^ Cells and Decreased Expression of PD1 in MDS Patients with AI

**Figure 1:**
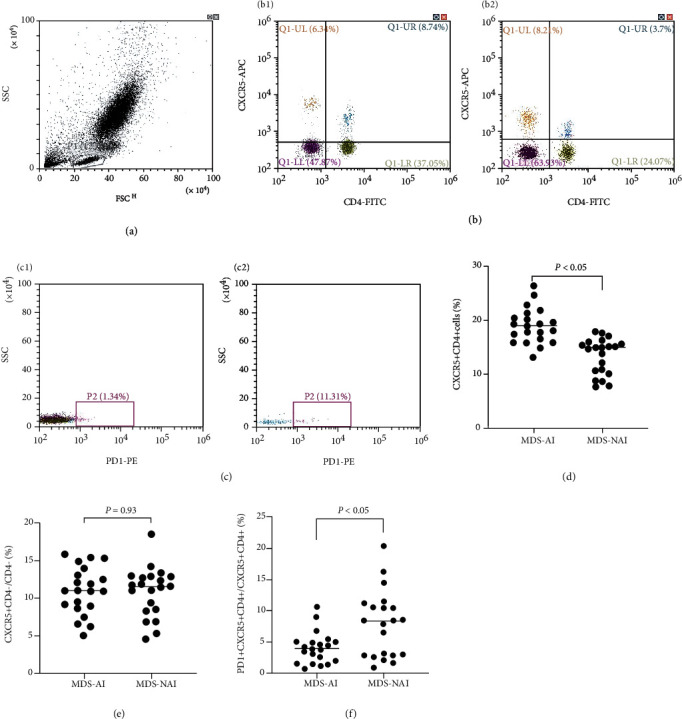
Increased frequency of CD4^+^CXCR5^+^ lymphocytes in the peripheral blood of newly diagnosed MDS patients with autoimmune disease (MDS-AI). (a) The gate of lymphocyte population in the peripheral blood with forward scatter (FSC) and side scatter (SSC). (b) Representative dot plots from flow cytometric (FACS) analyses showing the CD4^+^CXCR5^+^ lymphocyte frequency among peripheral blood obtained from newly diagnosed MDS-AI (B1) and newly diagnosed MDS patients without autoimmune disease (MDS-NAI) (B2). (c) Representative dot plots from FACS analyses showing the PD1 expression of CD4^+^CXCR5^+^ lymphocyte frequency among peripheral blood obtained from MDS-AI (C1) and MDS-NAIs (C2). (d) Frequency of CD4^+^CXCR5^+^ lymphocytes from peripheral blood was compared between MDS-AI (*N* = 21) and MDS-NAI (*N* = 21). (e) Frequency of CD4^−^CXCR5^+^ lymphocytes from peripheral blood were compared between MDS-AI (*N* = 21) and MDS-NAI (*N* = 21).(f) Frequency of PD1 on CD4^+^CXCR5^+^ lymphocytes from peripheral blood was compared between MDS-AI (*N* = 21) and MDS-NAI (*N* = 21). The bars represent the standard error of the mean.

The proportion of CD4^+^CXCR5^+^ cells in peripheral blood was significantly increased in MDS patients with AI (19.02 ± 3.23%) than in MDS patients without AI (13.46 ± 3.32%) (*P* < 0.05). However, there was no significant difference in the proportion of CD4^−^CXCR5^+^ cells between MDS patients with AI and without AI (11.01 ± 3.13% vs. 11.09 ± 3.26%, *P* > 0.05). The expression of PD1 on CD4^+^CXCR5^+^ cells was decreased in MDS patients with AI (4.09 ± 2.49%) than in MDS patients without AI (7.84 ± 5.28%) (*P* < 0.05) (Figure 1).

### 3.3. Serum IgG4 Levels Increased in MDS Patients with AI

**Figure 2:**
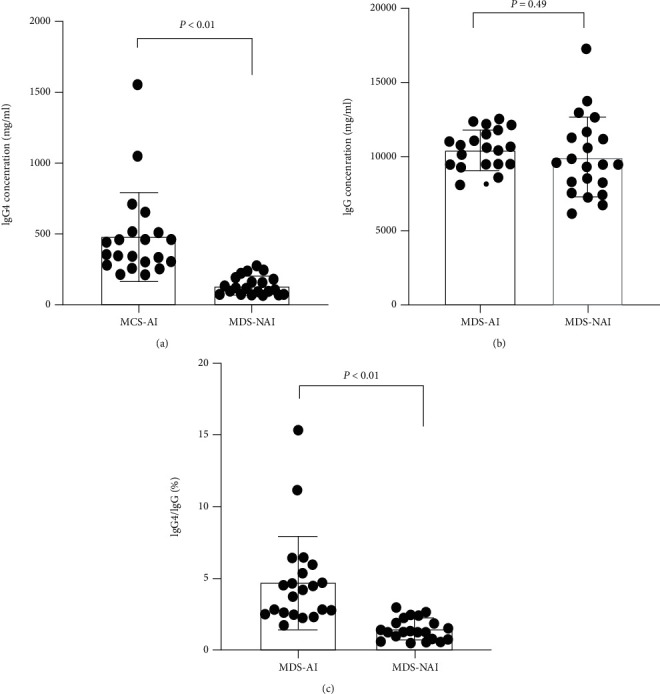
Elevated levels of IgG4 in serum from newly diagnosed MDS-AI. (a) The level of IgG4 in serum from newly diagnosed MDS-AI (*N* = 21) and MDS-NAI (*N* = 21). (b) The level of IgG in serum from newly diagnosed MDS-AI (*N* = 21) and MDS-NAI (*N* = 21). (c) The ratio of IgG4/IgG in serum from newly diagnosed MDS-AI (*N* = 21) and MDS-NAI (*N* = 21). The bars represent the standard error of the mean.

There was no significant difference in the level of serum IgG between MDS patients with AI and those without AI (10.418 ± 1.367 g/L vs. 9.963 ± 2.571 g/L, *P* > 0.05). The levels of serum IgG4 were significantly increased in MDS patients with AI (480.7 ± 312.3 mg/L) than in MDS patients without AI (139.9 ± 65.58%) (*P* < 0.05). The ratio of IgG4/IgG in the serum from MDS patients with AI was also higher (4.754 ± 3.228%) than that from MDS patients without AI (1.448 ± 0.738%) (*P* < 0.05) (Figure 2).

### 3.4. Outcome of Therapy

All patients with AI received glucocorticoid therapy, of whom 16 patients were treated with glucocorticoid alone and 5 patients with glucocorticoids combined with decitabine. The dosage of prednisone was 0.5-1.0 mg/kg/day or an equivalent dose of methylprednisolone. If glucocorticoid treatment was effective, it was continued. If it was ineffective within 4 weeks, the dose was rapidly decreased and eventually stopped. The dosage of decitabine (Janssen, China) was 20 mg/m^2^/day for 5 days, every 1 to 2 months according to the patient's conditions. MDS patients without AI were treated with recombinant human erythropoietin (Sansheng, China), lenalidomide (BeiGene, China) (only for the 5q- patient), decitabine (Janssen, China) (for MDS-EB), and support therapy.

According to the revised International Working Group (IWG) 2018 hematological response criteria [18], the overall response (OS) was 81% (17/21) in MDS patients with AI for the first-line treatment, including 75% (12/16) for glucocorticoids alone. Two patients had a complete response (CR), and three patients had a partial response (PR). Seven patients had a hematological response in erythroid cells (HI-E), four in platelets (HI-P), and one in neutrophils (HI-N).

### 3.5. Changes in Peripheral Blood CD4^+^CXCR5^+^ Cells and Serum IgG4 Level before and after Therapy

**Figure 3:**
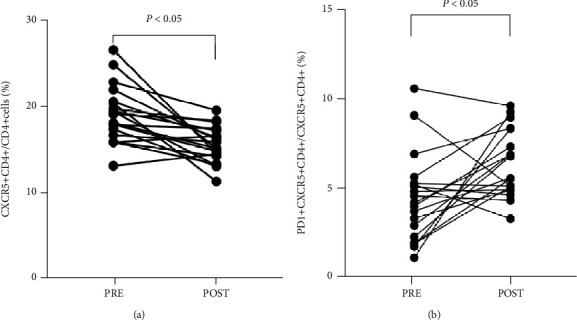
The change of CD4^+^CXCR5^+^ lymphocytes from newly diagnosed MDS-AI (PRE) and posttherapy (POST). (a) The quantity of CD4^+^CXCR5^+^ lymphocytes from newly diagnosed MDS-AI (PRE) and posttherapy (POST) (*n* = 19). (b) The PD1 expression of CD4^+^CXCR5^+^ lymphocytes from newly diagnosed MDS-AI (PRE) and posttherapy (POST) (*n* = 19).

Among the 21 MDS patients with AI, 19 patients had the data on CD4^+^CXCR5^+^ cells at pre- and posttherapy. The median interval time was 3 months (range 1-5 months). The proportion of CD4^+^CXCR5^+^ cells from MDS patients with AI decreased significantly (19.11 ± 3.22% vs. 15.59 ± 2.04%, *P* < 0.05) after treatment. Simultaneously, the expression of PD1 on CD4^+^CXCR5^+^ cells increased significantly in patients with AI after treatment (4.21 ± 2.52% vs. 6.21 ± 1.91%, *P* < 0.05) (Figure 3).

**Figure 4:**
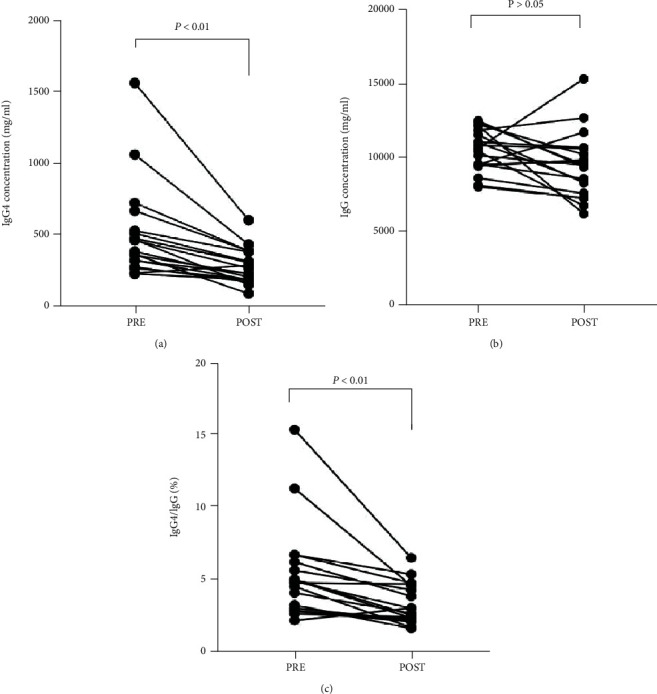
The change of serum IgG of newly diagnosed MDS-AI (PRE) and post-therapy (POST). (a) The change of serum IgG4 of newly diagnosed MDS-AI (PRE) and posttherapy (POST). (b) The change of serum IgG of newly diagnosed MDS-AI (PRE) and posttherapy (POST). (c) The fraction of serum IgG4/IgG of newly diagnosed MDS-AI (PRE) and posttherapy (POST) (*n* = 19).

There was no significant difference in the level of serum IgG between MDS patients with AI pre- and posttherapy (10.475 ± 1.411 g/L vs. 9.429 ± 2.197 g/L, *P* > 0.05). However, the level of serum IgG4 decreased significantly after treatment (498.1 ± 324.0 mg/L vs. 268.6 ± 121.3 mg/L, *P* < 0.05). The ratio of IgG4/IgG in serum from MDS patients with AI was also found to decrease significantly after treatment (4.96 ± 3.33% vs. 2.94 ± 1.42%, *P* < 0.05) (Figure 4).

### 3.6. Survival

**Figure 5:**
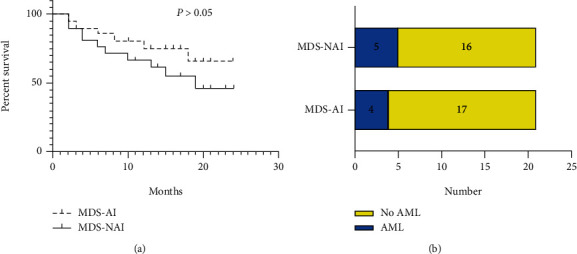
The survival time and transformation to AML of newly diagnosed MDS-AI and MDS-NAI. (a) The survival time of newly diagnosed MDS-AI and MDS-NAI (*P* > 0.05). (b) The transformation to AML of newly diagnosed MDS-AI and MDS-NAI (*P* > 0.05).

To verify the impact of AI on survival and AML progression, we compared MDS patients with AI (*n* = 21) and MDS patients without AI (*n* = 21). The median follow-up time was 15 months (range 2-24 months) in the MDS patients with AI group and 14 months (range 2-24 months) in the MDS patients without AI group. The median overall survival of patients with MDS with AI was not reached during the follow-up period. The survival rate of patients with MDS without AI was 19 months. There was no significant difference in overall survival between MDS patients with AI and MDS patients without AI (*P* > 0.05) (Figure 5).

Of the MDS patients with AI, four (19%) progressed to AML during the follow-up period, including three with acute monoblastic and monocytic leukemia (M5) and one with acute myelomonocytic leukemia (M4). Of the MDS patients without AI, five (23.8%) progressed to AML (*P* > 0.05), including three with acute monoblastic and monocytic leukemia (M5) and two with acute myelomonocytic leukemia (M4) (Figure 5).

## 4. Discussion

Studies have shown a link between MDS and autoimmune diseases. Approximately 11%-48% of patients with MDS coexisted with AI [5–8]. Patients with AI are significantly more likely to develop MDS and AML [19]. We diagnosed 107 MDS patients during the study period, of whom 21 (19.6%) had AI. These AIs included systemic vasculitis, systemic lupus erythematosus, rheumatoid arthritis, psoriasis, Sjogren's syndrome, inflammatory bowel disease, and Sweet's syndrome. Both relative low-risk patients (MDS-SLD and MDS-MLD) and high-risk patients (MDS-EB) can develop AI. The majority of MDS patients with AI respond for the first-line regimen based on glucocorticoids.

The specific mechanism of AI in MDS is unclear. Many studies have shown that the immune system is abnormal in MDS, including inflammatory cytokines and growth factors [20], toll-like receptors (TLRs) [21], mesenchymal stromal cells (MSCs) [22], monocyte-derived macrophages [23], dendritic cells [24], myeloid-derived suppressor cells (MDSCs) [25, 26], cytotoxic T lymphocytes, regulatory T cells (T regs) [27], and Th17 cells [28]. It is still uncertain whether B cells are derived from MDS clones, but there is evidence that B cell signals in MDS are abnormal [7]. Our previous studies have shown that Tfh cells are abnormal in low-risk MDS patients and mice [29, 30].

In this study, we found that the proportion of CD4^+^CXCR5^+^ cells increased in the peripheral blood of patients with MDS and AI, whereas the PD1 expression of the CD4^+^CXCR5^+^ cells decreased. PD-1 is an inhibitory B7-family molecule highly expressed on Tfh cells localized inside the germinal center territory. PD-1 controls Tfh cell tissue positioning and function [31]. Increased expression of PD-1 would therefore affect the localization and function of Tfh cells in tissues, including the helper function of B cells. Therefore, we believe that the increase in CD4^+^CXCR5^+^ cells and decreased expression of PD1 are related to the AI of patients with MDS.

We tested the serum IgG levels in patients with MDS and found that there was no difference in total IgG between the groups of MDS patients with and without AI, but the IgG4 level in the AI group was significantly increased. Tabata et al. [32] reported an MDS patient with autoimmune pancreatitis whose serum IgG4 levels were highly elevated. IgG4 has the lowest proportion of IgG subclasses in human serum at less than 4%. After repeated or long-term antigen stimulation, the IgG4 levels increase. The increase of IgG4 in tissues and serum has been found to be related to inflammation in a variety of chronic pathological conditions, such as IgG4-related diseases (IgG4-RD) [33] and rheumatoid arthritis [34]. IgG4 irregularities have also been reported in cancers, including melanoma [35], and pancreatic cancer [36]. In patients with tumors, the immune system is exposed to tumor-associated antigens for a relatively long period of time, and tumor cells promote inflammation. IgG4 may contribute to the escape of tumor cells from immune surveillance [37]. In patients with MDS, MDS clonal cells promoting inflammation and an inflammatory bone marrow microenvironment play essential roles in disease pathogenesis. Aberrant innate immune responses and proinflammatory signaling have been identified as key pathogenic drivers of MDS. In particular, S100A9-mediated NLRP3 inflammasome activation drives inflammatory conditions, leading to pyroptosis [38]. IgG4 may compete with IgG1 to bind to the target sites, inhibiting the immune surveillance function of IgG1.

In conclusion, we found that increased peripheral blood CD4^+^CXCR5^+^ cells and decreased expression of PD1 and increased IgG4 level in MDS patients with AI may be involved in the pathogenesis of MDS with AI. Targeting this irregularity may be an interesting direction for treatment of this type of MDS.

## Figures and Tables

**Table 1:** The characteristics of MDS patients with autoimmune diseases.

	MDS-AI	MDS-NAI	*P*
Number	21	21	
Age (years)	49 (20-87)	53 (21-83)	0.4503
Sex (male/female)	8/13	11/10	0.5359
MDS subtype			0.8596
MDS-SLD	3	4	
MDS-MLD	6	5	
MDS-RS	0	1	
MDS-EB1	7	6	
MDS-EB2	5	5	
IPSS-R			0.9370
Very low	2	3	
Low	5	5	
Intermediate	5	5	
Poor	4	5	
Very poor	5	3	
Karyotype			0.8874
Very good	1	0	
Good	14	14	
Intermediate	3	4	
Poor	2	2	
Very poor	1	1	
Median BM blasts (%)	5.5 (0-19)	6 (0-19)	0.9247

MDS-AI: MDS with autoimmune disease; MDS-NAI: MDS without autoimmune disease; BM: bone marrow.

**Table 2:** Different types of MDS-associated autoimmune disease.

Autoimmune disease	*n*
Systemic vasculitis	5
Systemic lupus erythematosus	4
Rheumatoid arthritis	3
Psoriasis	2
Sjogren's syndrome	2
Inflammatory bowel disease	1
Sweet's syndrome	1
Behcet's disease	1
Pyoderma gangrenosum	1
Granulomatosis with polyangiitis	1

## Data Availability

The data used to support the findings of this study are included within the article.

## Supplementary Materials

Supplementary Materials Sample collection, preparation, gate, and analysis.
