# Supplementary material for: Increased Circulating CD4+CXCR5+ Cells and IgG4 Levels in Patients with Myelodysplastic Syndrome with Autoimmune Diseases
Source: J Immunol Res. 2021 Sep 30;2021:4302515. doi: 10.1155/2021/4302515 (PMC8497158; doi:10.1155/2021/4302515)
Supplement: Supplementary Materials — Sample collection, preparation, gate, and analysis. [file 4302515.f1.docx]

***Sample collection, preparation, gate and analysis:***

Peripheral blood samples were obtained by standard venipuncture using disposable venous blood lancets and vacuum blood tubes.

FITC-CD4, APC-CXCR5, PE-PD1 and isotype controls monoclonal antibodies were purchased from BD Biosciences. All antibodies were titrated to saturating concentrations and the corresponding isotype controls were prepared. Diluted blood was stained and incubated in the dark at room temperature for 15-20 minutes. Flow cytometric measurements were performed within 30 minutes after the samples were obtained. Data acquisition and analysis were performed using a FACS-Calibur flow cytometer (BD Biosciences, USA) and Cell Quest software (Becton Dickinson, version 3.1).

We used cells’ forward scatter (FSC) and side scatter (SSC) to divide peripheral blood mononuclear cells into three subgroups, namely lymphocytes (P1 in figure A), monocytes, and granulocytes.


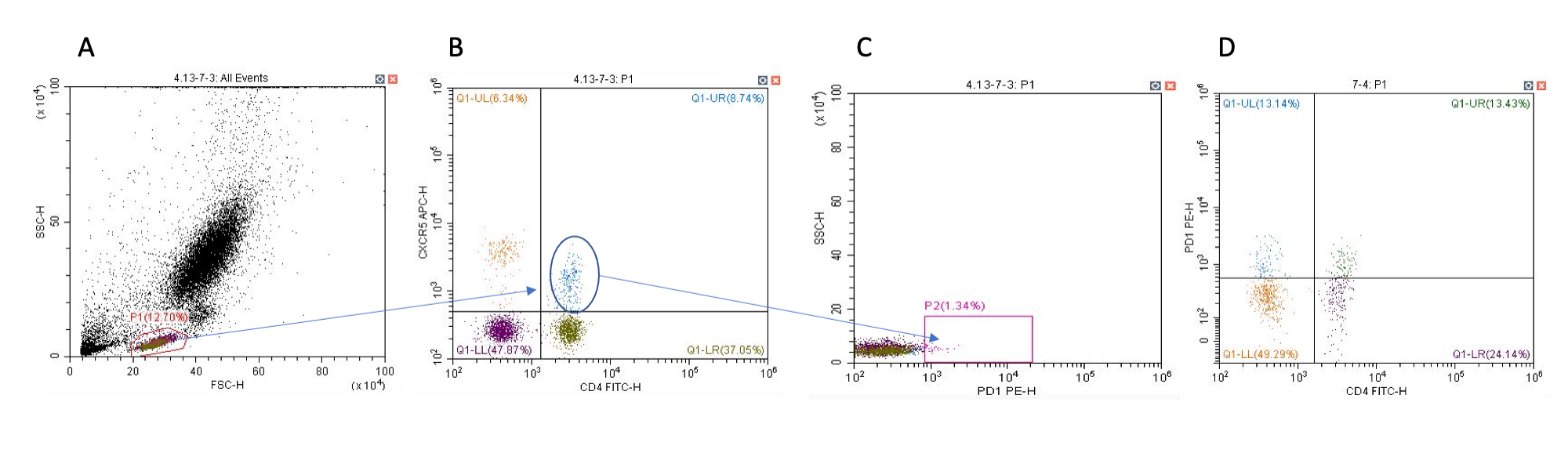


Then we gated the CD4+CXCR5+ lymphocytes (Q1-UR in figure B) and detected the expression of PD1 on CD4+CXCR5+ lymphocytes (P2 in figure C). The expression of PD1 on CD4+ lymphocytes (Q1-UR in figure D)

The representative dotplots of CXCR5 vs PD-1 for CD4+ T cells were in figure B and D.
